# An IRT–Multiple Indicators Multiple Causes (MIMIC) Approach as a Method of Examining Item Response Latency

**DOI:** 10.3389/fpsyg.2018.02177

**Published:** 2018-11-13

**Authors:** Ioannis Tsaousis, Georgios D. Sideridis, Abdullah Al-Sadaawi

**Affiliations:** ^1^Department of Psychology, University of Crete, Rethymno, Greece; ^2^Institutional Centers for Clinical and Translational Research, Boston Children's Hospital, Harvard Medical School, Boston, MA, United States; ^3^Department of Primary Education, National and Kapodistrian University of Athens, Athens, Greece; ^4^Psychology Department, College of Education, King Saud University, Riyadh, Saudi Arabia; ^5^National Center for Assessment, Riyadh, Saudi Arabia

**Keywords:** item response latency, computer based testing (CBT), educational testing, multiple indicator multiple causes model (MIMIC), IRT-MIMIC

## Abstract

The analysis of response time has received increasing attention during the last decades, since evidence from several studies supported the argument that there is a direct relationship between item response time and test performance. The aim of this study was to investigate whether item response latency affects person's ability parameters, in that it represents an adaptive or maladaptive practice. To examine the above research question data from 8,475 individuals completing the computerized version of the Postgraduate General Aptitude Test (PAGAT) were analyzed. To determine the extent to which response latency affects person's ability, we used a Multiple Indicators Multiple Causes (MIMIC) model, in which every item in a scale was linked to its corresponding covariate (i.e., item response latency). We ran the MIMIC model within the Item Response Theory (IRT) framework (2-PL model). The results supported the hypothesis that item response latency could provide valuable information for getting more accurate estimations for persons' ability levels. Results indicated that for individuals who invest more time on easy items, their likelihood of success does not improve, most likely because slow and fast responders have significantly different levels of ability (fast responders are of higher ability compared to slow responders). Consequently, investing more time for low ability individuals does not prove to be adaptive. The opposite was found for difficult items: individuals spending more time on difficult items increase their likelihood of success, more likely because they are high achievers (in difficult items individuals who spent more time were of significantly higher ability compared to fast responders). Thus, it appears that there is an interaction between the difficulty of the item and person abilities that explain the effects of response time on likelihood of success. We concluded that accommodating item response latency in a computerized assessment model, can inform test quality and test takers' behavior, and in that way, enhance score measurement accuracy.

## Introduction

The use of Computer Based Tests (CBT) in educational and psychological assessment has becoming increasingly popular in recent years. This method of test administration has given the opportunity to test developers to elicit important information regarding the individuals' reactions toward test items but also regarding the impact of the items on the individuals' performance (e.g., Verbić and Tomić, 2009; Ranger and Kuhn, 2012). One of the most useful recorded information during a CBT administration is response time or response latency. According to Zenisky and Baldwin (2006), we can distinguish between two forms of response latency: *item response latency* (how long it takes for a test-taker to answer an item) and *test response latency* (how long it takes for a test-taker to complete the whole test (Lee and Chen, 2011). Previous research has shown that the consideration of response latency increases the precision of performance estimation (Wang and Hanson, 2005; Ranger and Kuhn, 2012). Apart from this, previous research has also shown that response time at item or test level could be used for several other purposes such as: to select items in the context of Computerized Adaptive Testing (CAT) (e.g., Wang and Hanson, 2005; van der Linden, 2010); to identify aberrant items (van der Linden and van Krimpen-Stoop, 2003); to enhance the construct validity of the measure, by taking into account construct-irrelevant variances caused by factors (e.g., speededness) that are not intentionally part of the construct being measured (Zenisky and Baldwin, 2006); to determine the optimum time limit on tests (Halkitis et al., 1996); Finally, such information could be used as an additional indicator for detecting faking behavior in non-cognitive tests, since previous findings suggest that lying is associated with longer response latencies (Holden and Kroner, 1992).

Recent findings have also shown that response latency could provide valuable diagnostic information in terms of both, the test's quality as well as the test-taker's performance (van der Linden, 2009). For example, longer items in terms of the number of words, the number of clauses or sentences, and the number of response options take longer to answer. Similarly, items requiring extensive cognitive processes (e.g., complex mathematical operations) or open-ended questions are subject to longer processing times (Yan and Tourangeau, 2008). On the other hand, there is a vast amount of empirical evidence suggesting that response latency is an important factor in human decision processing (Ranger and Kuhn, 2012). Particularly, in the field of experimental psychology, Thomas et al. (2008) found that when the given alternatives in an item are becoming increasingly similar, higher levels of mental effort are required by the individual to solve the problem, which in turn, leads to longer response time. Finally, people with a lower level of cognitive ability, older people, people with cognitive impairments or people with less education are found in need of more time to come up with answers (Yan and Tourangeau, 2008; Couper and Kreuter, 2013).

Another line of research has examined the relation between response latency and response accuracy (e.g., Bolsinova et al., 2017a,b). Particularly, Klein Entink et al. (2009a) and Goldhammer and Klein Entink (2011) found that there is a positive correlation between slowness and correct answers in reasoning tests. The same pattern of results was reported in a study where the relationship between slow response time and ability of complex problem solving was examined (Scherer et al., 2015). On the other hand, Goldhammer et al. (2013) reported a negative correlation between slowness and basic computer skills, while van der Linden et al. (1999) found that there is zero correlation between slow response time and arithmetic ability. Finally, González-Espada and Bullock (2007) reported that there is a significant difference between the average response latency for items answered correctly and incorrectly, with correctly answered items requiring less response time.

Other scholars have examined the role of response latency on performance accuracy. In other words, whether the time a test-taker spends on an item affects precision in estimating this person's ability parameters (see Schnipke and Scrams, 2002, for an overview of the literature). Wang and Hanson (2005) examined the effect of response latency on item parameter estimation using a 4-PL Item Response Theory (IRT) model, in which response latency was incorporated as the fourth parameter (the item and person slowness parameter). In this study, the slowness factor was treated as a fixed predictor rather than a random effect variable. The results obtained from both, real and simulated data, showed that response latency affects the correct answer probability, and that ignoring response latency in parameter estimation will have an adverse effect in estimating examinee's ability.

Halkitis et al. (1996) also investigated whether response latency has a direct effect on item parameters. Particularly, they examined whether response latency is related item difficulty, item discrimination, the point-biserial correlations, and length of the item. They reported that as item difficulty, item discrimination, and length of the item increase, response latency also increases. In another study, Smith (2000) examined the relationship between item response latency and item characteristics (i.e., item difficulty, item discrimination, figure in an item, and length of the item across different cognitive domains). The most noticeable finding was the positive relationship between item difficulty and response latency, a relationship that was found to be consistent across different cognitive domains, such as problem solving, data sufficiency, sentence correction, critical reasoning, and reading comprehension.

Bridgeman and Cline (2000) examined the effect of response latency on five levels of item difficulty (from very easy to very difficult), on different item types (i.e., problem solving vs. quantitative comparison), on different cognitive domains (i.e., arithmetic, algebra, geometry, and data interpretation), and on degree of abstraction (numbers and symbols vs. word texts). They replicated the finding that item difficulty is strongly and positively related to response latency, with more difficult items requiring more time. Interestingly, they reported large amounts of variability in response latency across individuals on items of equal difficulty levels and similarity in content Finally, they found that for items with long expected response time due to their format (e.g., text items), longer response latencies were not associated with overall performance levels. In a similar study, Yang et al. (2002) found a positive relationship between item difficulty and response latency across slow and fast respondents. They reported that slow responders tend to spend significantly more time than fast responders, and that for more difficult test items test-takers usually need more time to respond.

Parshall et al. (1994) examined whether response latency is affected by several item characteristics, including presentation order, content classification, and cognitive classification. They found that the first two factors were related to response latency, although cognitive classification was not. Finally, Masters et al. (2005) investigated the relationship between item difficulty level and response latency as well as the skill to do calculations or the need to involve external supplemental information on response latency. They found that response latency is mutually affected by item difficulty and the content of the item.

How person's individual characteristics (e.g., gender, age, race, etc.) may affect item response latency has also been examined. For example, Schmitt et al. (1991) probed for differences in response latency across gender. They concluded that gender does not affect the relationship between response latency and test performance. Cole (1997), replicated the findings of Schmitt et al. (1991) by use of a meta-analytic study, which involved data from 400 tests and millions of test-takers. In another study, Yan et al. (2015) examined the effect of response latency across slow and fast respondents at different age groups. They found that for respondents aged between 50 and 70, the longer the time a test-taker spends on answering an item the lower the quality of the response. However, the opposite was true for respondents aged 70 and above: the longer the time spent on answering an item the higher the quality of the response, pointing to the moderating role of developmental differences.

Schmitt and Bleistein (1987) found significant differences between racial groups on test response latency with Caucasian test-takers responding faster than African Americans. In another study, Schmitt et al. (1991) reported significant differences in item response times on Scholastic Aptitude Test (SAT) among African American, Hispanic, and Caucasian test-takers, with Caucasian individuals responding faster compared to any other group. The same results were replicated by Lawrence (1993), who examined item response latency across different ethnic groups on the Graduate Management Admission Test (GMAT). Another personal characteristic that was found to influence response latency (at either the test or item levels) is test-takers' anxiety level. Bergstrom et al. (1994) found that anxiety was significantly related to test response latency and test performance. Particularly, they found that anxiety levels moderate the relationship between response latency and test performance, with more anxious test-takers in need of additional time to answer an item correctly.

Another interesting line of research in the response latency literature is the role that idiosyncratic characteristics such as speededness (i.e., spontaneous and fast vs. thoughtful and slow responses) play during testing. Previous findings from Psychology and Education (but also in other research areas such as public opinion research), suggest that differences in temperament influence test performance (Mayerl, 2013). For example, Yang et al. (2002) found that slow responders tend to spend significantly more time compared to fast responders on the most difficult items/sub-tests. They also found a significant positive relationship between item difficulty and response latency across slow and fast respondents, with slow respondents needing more time to respond. According to Kennedy (1930), there are distinct characteristics between fast and slow types of respondents: “The slow type is supposed to plod along persistently with great care for details and accuracy. The quick type, …, works in a more slap-dash fashion, has little regard for details, and is inclined to be inaccurate” (p. 286). However, there are several studies in which the results suggest the opposite. For example, Hornke (2005) found that higher response latencies are associated more with incorrect rather than correct responses, since individuals who do not know an answer usually spend more time trying to find (or even guess) the correct answer but with limited success. Similar findings have been reported from other scholars as well (e.g., Swanson et al., 2001).

In the extant literature, two approaches in the investigation of response latency on testing performance have been proposed. The first examines response latency within the IRT framework, where response latency is incorporated in the item response model. For example, Wang and Hanson (2005) proposed a variation of the 3PL model, called the four-parameter logistic RT (4PLRT) model. In this model, response latency is part of the item response model, since it is treated as a fixed predictor rather than a random variable. This method is considered as more sophisticated, since it assumes that there is an interaction between the parameters that govern the distributions of the person's reaction time and their response on the items (van der Linden, 2006). Comparable methodologies can be found in Wang (2006); Lee (2007); van der Linden (2007); Klein Entink et al. (2009a), and Meyer (2010) but also in earlier attempts, such as Roskam's (1997), Thissen's (1983), van Breukelen's (1989), and Verhelst et al. (1997). In the second approach, response latency is modeled independently of the response variables for the items. In other words, response time distributions are modeled without any parametric relation to the distribution of the response variables on the items. Typical examples of this approach are found in Maris (1993), Scheiblechner (1979), Schnipke and Scrams (1997), van der Linden et al. (1999), and van der Linden and van Krimpen-Stoop (2003). It should be noted, herein, that this overview on the different methods on response latency literature is not exhaustive, since only a few paradigms from each approach have been selected. A more thorough review of the history of response latency analysis can be found in Schnipke and Scrams (2002).

The current study was prompted by the fact that empirical findings suggest that response latency is an important factor in testing process, since it could provide valuable information for getting more accurate estimations for persons' ability levels. Thus, this study could be viewed as part of the growing body of research on the extent to which response latency affects test's characteristics and person's performance. To examine that, we introduce the IRT–Multiple Indicators Multiple Causes (MIMIC) model in this line of research, to provide further insight into the relationship between test items, examinee response time, and examinee performance. Although, the IRT–MIMIC model represents a well-established methodology in psychometrics, its application in this line of research is very limited. We propose that the IRT–MIMIC model can easily be adapted to investigate and control for the effects of response latency during testing. To this perspective, the current study was prompted to examine the extent to which item response latency affects person's ability parameters, and as a result, provides more accurate estimation of test-takers' performance by use of the IRT–MIMIC model.

## Materials and methods

### Participants and procedure

A total of 8,475 individuals from different places of the Kingdom of Saudi Arabia participated in this study. From them, 4,201 (49.6%) were males and 4,274 (50.4%) were females. In terms of place of residence, participants came from all 13 provinces of the Kingdom of Saudi Arabia, with the majority of them coming from the urban cities of Riyadh, Makkah, and Eastern Province. No other demographic information was available. The data were collected from January to December of 2015 and all participants completed the computerized version of the Postgraduate General Aptitude Test (PAGAT). The study was conducted as part of a National Examination in Saudi Arabia meeting ethical approval standards from the National Center for Assessment in Higher Education (Qiyas) Ethics Committee. All participants were informed that their responses would be utilized as part of a larger study to evaluate the psychometric properties of the measure. Completion of the test comprised their informed consent for their participation. No participants reported any psychological ore emotional issues that would inhibit their full performance.

### Measure

*The General Aptitude Test for Postgraduate Students* (GAT-Post; National Center for Assessment in Higher Education-NCA). This is a 104-item test, which measures a university graduate's analytical and deductive skills. It focuses on testing student's capacity for learning in general regardless of any specific skill in a certain subject or topic. There are three major cognitive areas: (a) verbal, (b) quantitative, and, (c) advanced functioning. The Verbal domain is composed of four scales: Analogy, Sentence Completion, Context Analysis, and Reading Comprehension. The Quantitative domain consists of three scales: Arithmetic, Analysis, and Arithmetic Comparisons. Last, the Advanced Functioning domain consists of three scales: Critical Thinking, Spatial, and Logic. This test has 2.5 h duration, and all participants should respond to all items within the time frame.

### Data analyses

Structural Equation Modeling (SEM) analyses were conducted using Mplus Version 8 (Muthén and Muthén, 1998-2016). Due to the binary nature of the data (1 = correct, 0 = erroneous responding), the mean and variance-adjusted weighted least squares (WLSMV) estimation method was used (Muthén et al., 1997). This estimator is well-suited when multivariate normality assumptions cannot be guaranteed (Brown, 2015). First, a Confirmatory Factor Analysis (CFA) model was conducted in order to establish a statistically acceptable model for each examined PGAT scale. A unidimensional model was assumed for all scales based on the theoretical framework provided by the developers of the test (NCA). Model fit was evaluated by fitting the data to a tetrachoric correlation matrix using the probit link function. Model fit was evaluated by use of three fit indices that showed good performance in a simulation study by Hu and Bentler (1998): The *Root Mean Squared Error of Approximation* (RMSEA), the *Comparative Fit Index* (CFI), and the *Tucker-Lewis Index* (TLI), also termed as the Non-Normed Fit Index (NNFI). For the RMSEA, values < 0.08 are indicative of good model fit, with values < 0.05 suggesting excellent fit (Hu and Bentler, 1999). For the remaining two fit indices, values >0.90 indicate acceptable model fit (with values >0.95 being ideal; Hu and Bentler, 1999).

After evaluating the measurement model for each PGAT scale, a MIMIC model was used to examine the effect of response latency on the individual items for each scale. The MIMIC model is a special form of SEM that integrates causal variables (i.e., covariates) with a CFA (MacIntosh and Hashim, 2003). Each MIMIC model is composed of two parts: a measurement model and a structural model. The first examines the relations between a latent variable and its indicators (observed variables); the latter examines the effect of the covariates on factors and/or factor indicators, thereby estimating the effect of covariates on latent factor means and/or item parameters, respectively (Jöreskog and Sörbom, 1996). One of the main advantages of this model is that it allows for the evaluation of the effect of the covariates on each factor indicator, and simultaneously, all obtained estimates are adjusted for the effects of all the covariates in the model (Muthén et al., 1991; Brown, 2015).

MIMIC models have mainly been used in the literature as a method for detecting Differential Item Functioning (DIF) (Finch, 2005; Woods, 2009; Wang, 2010; Woods and Grimm, 2011), although some scholars have argued that this approach is not always very effective in identifying DIF (e.g., Teresi, 2006; Chun et al., 2016; Lee et al., 2017). Based on Dorans and Holland (1993): “*DIF refers to differences in item functioning after groups have been matched with respect to ability or attribute that the item purportedly measures … DIF is an unexpected difference among groups of examinees who are supposed to be comparable with respect to attribute measured by the item and the test on which it appears*” (p. 37). However, several scholars have argued that MIMIC models can be utilized for investigating the examination of a more comprehensive relationship between the covariate(s) and both, the latent factor as well as the factor indicators (e.g., Muthén, 1988).

As mentioned above, the MIMIC model can also include direct effects of the covariates on indicators, holding the latent variables constant or also estimate indirect effects via the factor. These direct paths can examine possible differential responding of the item at different levels of the covariate (i.e., item response latency), that is, DIF. The model tests the probability that item *u*_*j*_ that belongs to factor η_*i*_ and receives a direct effect from a dichotomous covariate *x*_*i*_ (i.e., response latency) has a response probability of 1 as shown below (Muthén, 1989):

(1) $$\begin{array}{l} u_{ij}^{*}=\lambda_{j}\eta_{i}+\kappa_{j}x_{ij}+\varepsilon_{ij} \end{array}$$

with λ being the factor loading of item *j* on factor η with a mean of zero, κ_*j*_ being the effect of the covariate on item *u*_*j*_ at values of response time *x*_*i*_. The probability of correct responding is then estimated as follows:

(2) $$P\left(u_{ij}=1|\eta_{i},x_{ij}\right)=1-F\left[(\tau_{j}-\lambda_{j}\eta_{i}-\kappa_{j}x_{ij}\right)\frac{1}{\sqrt{\theta_{jj}}}],$$

With θ_*jj*_ being the item residual variance, τ_*j*_ the item threshold, λ_*j*_ the factor loading, η_*i*_ the factorbeing estimated, κ_*j*_ the effect of the covariate *x*_*ij*_ (specific response time *i* of item *j*), and *F* the normal distribution function (Muthén et al., 1993).

**Figure 1:**
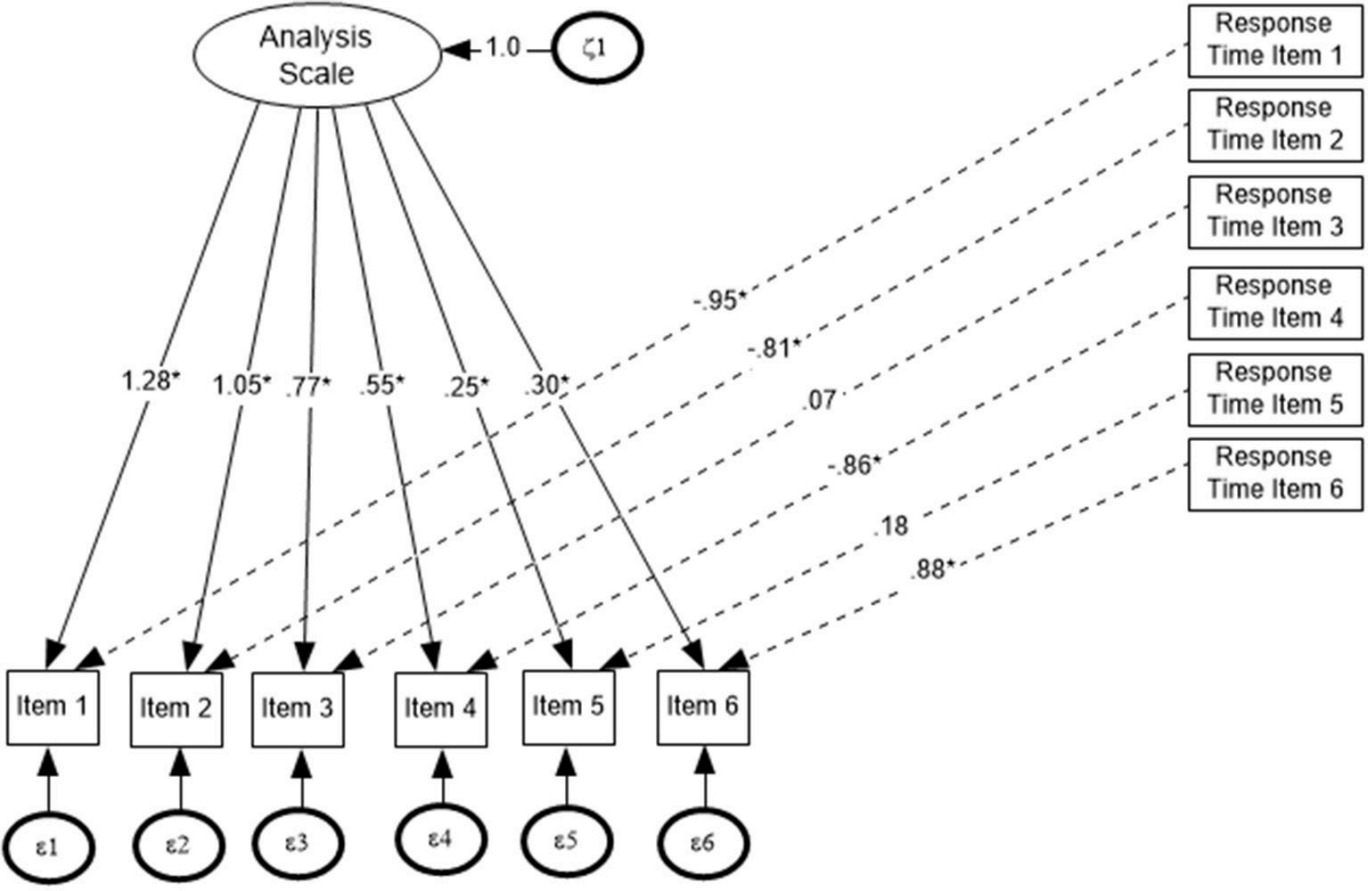
The IRT–MIMIC model for Analysis (ANA) scale.

For the implementation of the MIMIC model, robust maximum likelihood [Maximum Likelihood with robust standard errors (MLR)] was used as the estimation method. MLR provides a chi-square test statistic that is robust to non-normality and non-independence of observations. In that regard, the MIMIC model threshold parameters can easily be transformed to the respective estimates of a 2-parameter IRT model (e.g., MacIntosh and Hashim, 2003). In Figure 1, the generic IRT–MIMIC model is shown, in which every item was linked to its corresponding item response latency.

## Results

Descriptive statistics and inter-correlations among the scales of the PGAT are shown in Table 1. Aimed at demonstrating the benefits of the MIMIC–IRT approach for examining the role of response latency on persons' ability, the results from the Analysis (ANA) subscale of the quantitative domain are shown in the main text's Results section. Results from the remaining subscales can be found in Supplementary Material that accompanies this manuscript.

**Table 1:** Descriptive statistics and inter-correlations among the study variables.

	**Mean**	**SD**	**1**	**2**	**3**	**4**	**5**	**6**	**7**	**8**	**9**	**10**	**11**	**12**	**13**	**14**
1. AN	9.59	2.83														
2. SC	4.24	1.29	0.35													
3. CA	5.78	2.05	0.39	0.34												
4. RC	10.90	3.08	0.49	0.38	0.44											
5. AR	4.37	1.71	0.28	0.23	0.26	0.30										
6. ANA	3.44	1.29	0.33	0.20	0.25	0.33	0.19									
7. CO	3.54	1.36	0.38	0.26	0.31	0.39	0.22	0.43								
8. CT	7.81	2.45	0.39	0.23	0.32	0.46	0.24	0.40	0.45							
9. SP	6.65	1.63	0.37	0.23	0.28	0.41	0.22	0.38	0.42	0.58						
10. LG	4.63	2.00	0.39	0.26	0.31	0.45	0.24	0.37	0.45	0.47	0.42					
11. Verb	30.52	7.03	0.80	0.59	0.70	0.84	0.37	0.39	0.46	0.49	0.45	0.49				
12. Num	11.35	3.13	0.46	0.32	0.38	0.47	0.72	0.70	0.73	0.49	0.46	0.48	0.56			
13. Advnc	19.09	4.97	0.47	0.29	0.37	0.54	0.29	0.47	0.54	0.87	0.78	0.76	0.59	0.59		
14. PGAT	60.96	12.96	0.72	0.51	0.62	0.77	0.48	0.56	0.64	0.72	0.66	0.68	0.90	0.77	0.85	

*All correlation coefficients were significant at p < 0.001 level; AN, Analysis; SC, Sentence Completion; CA, Context Analysis; RC, Reading, Comprehension; AR, Arithmetic; ANA, Analysis; CO, Arithmetic Comparisons; CT, Critical Thinking; SP, Spatial; LG, Logic; Verb, Verbal, Overall Score; Num, Numeric Overall Score; Advnc, Advance Maths Overall Score*.

As expected, all scales within each domain were moderately positively correlated. Furthermore, all scales within a domain were highly inter-correlated with their corresponding domain overall scores (in bold). In Table 2, the minimum, maximum, and average response latencies (in s) for the Analysis scale are shown. For item 2, the most difficult item in the scale, the maximum response time invested was 421 s. In contrast, the respective response time for the easiest item (item 4) was 201 s.

**Table 2:** Minimum, maximum, and average response latencies (in s) for each item of the Analysis (ANA) scale.

**Item**	**Minimum**	**Maximum**	**Average**	**Standard deviation**
1	2	201	29.77	20.96
2	2	247	38.85	24.88
3	2	416	55.21	36.72
4	1	329	51.65	35.64
5	2	354	51.28	34.88
6	2	421	37.87	25.61

Next, we ran a CFA model to verify the hypothesized unidimensional structure of the Analysis scale. Results showed excellent model fit, suggesting a close approximation of the data to the model [χ^2^_(9, N = 8, 475)_ = 20.91, *p* = 0.0130; RMSEA = 0.012 (90% CI = 0.005–0.020); CFI = 0.991, TLI = 0.985]. It should be noted here that the chi-square test as a measure of global model fit is hypersensitive to sample size, since it rejects reasonable models whenever sample sizes are large and it fails to reject poor models whenever sample sizes are small (MacCallum et al., 1996). Furthermore, it is well known that the chi-square test is an inherently flawed mechanism for addressing model fit, especially in terms of comparing the observed chi-square statistic to a chi-square distribution because this test is not robust to violations of the distributional assumptions and because the distribution itself provides only asymptotically correct *p*-values (i.e., the *p*-value approaches its correct value as the sample size becomes infinitely large). For those reasons, and in the presence of a large sample size in the present study and the associated excessive levels of power, we deferred from further utilizing the chi-square test (Brown, 2015).

Next, we examined whether item response latency had an effect on items' thresholds. Prior to that, however, it was important to examine the Wang and Hanson (2005) assumption, in that response time should be independent of the person's ability, when someone investigates the role of response latency on person's performance. This is a necessary prerequisite assumption, in order to ensure that the outcome of this study is not simply a statistical artifact. The results from the analysis showed that the correlation between the person's theta and individual item's response latency for the Analysis scale ranged between 0.01 (item 1) and 0.26 (item 2) confirming that response time was independent of persons' ability.

Next, the effect of response latency on person's ability was examined using an IRT–MIMIC approach, using as covariates the response latencies for each item^1^. The probability of success, and, respective ability level in logits, for groups classified as fast vs. slow responders (based on the covariate), are shown in Table 3. It should be noted that for this analysis, and in order to align the MIMIC models' findings to that of a DIF analysis, response latency was recoded as a binary variable based on a z-score transformation. Values of zero represented response times lower than the mean and values of one, greater than the mean on the z-variate.

**Table 3:** Probability of success and person's estimated ability (in logits) for each item of the Analysis (ANA) scale across two groups of respondents defined by speed of response.

	**Fast respondents**		**Slow respondents**	
**Item**	**Probability of success (%)**	**Person's ability (in logits)**	**Probability of success (%)**	**Person's ability (in logits)**
1	98	4.06	80	1.40
2	92	2.48	67	0.72
3	93	2.57	92	2.40
4	48	−0.08	16	−1.63
5	28	−0.93	35	−0.63
6	02	−3.88	13	−1.89

*Probability of success was estimated using Equation (2). Person ability in logits was estimated using the following formula: Person Ability = Log(P)/)1 – Log(P), where P is the probability of success as estimated using Equation (2)*.

The results from the analyses showed that there was a significant effect of response latency on all items of the Analysis scale. As shown in Table 3, for easy items (i.e., items 1 and 2), and for individuals who do not invest much time to answer the item, simply because they know the answer (i.e., fast respondents), the probability of success is almost 100%. Accordingly, their ability level is very high. However, for test-takers who need more time, because of uncertainly and lack of knowledge about the correct answer (i.e., slow respondents), the probability of succeeding is significantly reduced. Estimates of person abilities for fast vs. slow responders on easy items suggested that slow responders were of significantly lower ability (compared to fast responders). Consequently, the extra invested time by individuals of lower ability was not associated with respective achievement gains, likely because these individuals do not possess the necessary resources and knowledge base to answer the item correctly. When difficult items were encountered (e.g., items 5 and 6), the relationship between response latency and achievement was reversed. That is, for individuals who spend more time on difficult items, the probability of success was significantly elevated compared to individuals who responded quickly (fast responders). This was likely due to the fact that slow responders in difficult items represented high achieving individuals. Thus, ability level moderates the relationship between response latency and success with the additional time benefiting high ability individuals but not low ability test-takers. Investing additional time for high achieving individuals increases significantly their probability of success as the additional time likely utilizes resources that are available and necessary for deciphering the correct response.

**Figure 2:**
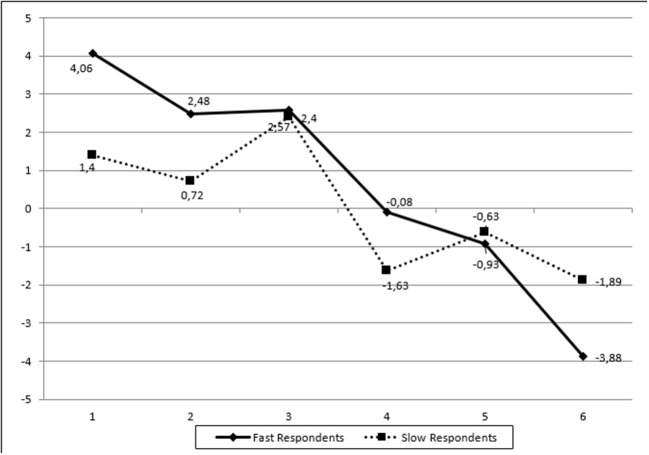
Person's estimated ability (in logits) across different groups of respondents (fast vs. slow) for the Analysis (ANA) scale.

Two additional pieces of information seem to inform the above conclusion. First, when the effects of additional time were beneficial (for high achievers on difficulty items), they represented large amounts of time compared to when additional time was not beneficial (for low achievers on easy items). That is, groups were classified as pending additional time (slow responders) when the maximum response latency was 200 s (on easy items) or 400 s (on difficulty items). Thus, the classification of individuals as utilizing additional time (i.e., slow responders) was item difficulty sensitive in that an individual would be classified as utilizing more time on an easy item (slow responder) and the same amount of time spent when viewed under the lens of a difficulty item would result in the classification of that person as a fast responder. In other words, when response latency was found to be adaptive, it involved difficult items and salient amounts of time. The second piece of information that aids the above interpretation comes from an investigation of the number of individuals classified as fast or slow responders. The concordance between those classifications for one item in relation to another item ranged between 30 and 60%. Thus, individuals were not classified as fast or slow responders across the board (i.e., test-takers were either fast or slow across all items). This type of information also explains the differences in mean level of ability between e.g., slow responders across items. A graphical representation of the above results is presented in Figure 2.

## Discussion

The aim of this study was to investigate whether item response latency affects person's ability parameters, in that it represents an adaptive or maladaptive practice. To determine the extent to which response latency affects person's ability, we used a MIMIC model within the IRT framework (2-PL model), in which every item in a scale was linked to its corresponding item response latency as a covariate. A MIMIC model provides a better insight into the relations between items, latent variables and covariates, by allowing the simultaneous evaluation of the effect of the covariates on the factor indicators. In that way, all the obtained estimates are adjusted for the effects on the covariates in the model, providing better estimation (Muthén et al., 1991; Brown, 2015).

First, we examined the measurement model for the examined scale. This is a prerequisite when a MIMIC model is applied. The results from the CFAs revealed that the measurement model (i.e., factor structure) of the scale had excellent fit indices, providing robust evidence for its unidimensional nature. Next, the effect of the response latency on each item was examined. The results from the MIMIC model showed that high response latency is indeed related to the probability of answering an item correctly, and provided further support to findings from previous studies, in which it was found that response latency affects the correct answer probability (e.g., Schnipke and Scrams, 2002; Klein Entink et al., 2009b; Goldhammer and Klein Entink, 2011). However, in the present study, response latency was conditional on item difficulty and person ability. That is, for low ability test-takers, the investment of additional time, on easy items, was not associated with enhanced likelihood of success. This may likely be due to the fact that the necessary cognitive and self-regulatory resources to attain positive achievement outcomes are unavailable for low achievers, thus, the additional time does not result in associated benefits. This finding agrees with a series of studies that reported a negative association between response latency and correct responding (e.g., Bergstrom et al., 1994; Swanson et al., 2001; Hornke, 2005). In support of the above finding, Hornke (2005) reported that higher response latencies were associated with incorrect responses, since low ability individuals may spend more time to decide on a guess or even cheat.

On the other hand, individuals who invested more time to answer difficult items (representing high ability test-takers), increased their likelihood of success. Thus, for high achieving individuals the additional time is beneficial as it likely is implemented to energize cognitive resources, to eliminate incorrect distractors, and make informed judgements that lead to the correct response. When looking at low response times in difficult items, that responder group had significantly lower ability levels. Thus, fast responding likely represents, absence of any prerequisite knowledge to attain correct responding and may even reflect non-attempts (i.e., choosing to skip item overall). This finding is in line with findings from past studies, not only from the domain of ability testing, but also from other domains, such as personality measurement (Ferrando, 2006), and other scientific fields, including psychophysics (e.g., Espinoza-Varas and Watson, 1994) and cognitive experimental psychology (Thomas et al., 2008), in which the likelihood of a correct answer increases as the time invested on an item also increases (Luce, 1986). The present study, however, informs the literature of a significant interaction between response latency and level of ability in that the additional time is beneficial only for high achieving individuals but has no added value for low achieving individuals.

Although the findings from this study could be considered as part of the growing body of research on the role that response latency plays on item's characteristics and person's estimated ability, this topic needs further exploration. For example, other factors such as the length of the item (Smith, 2000), the presentation order (Parshall et al., 1994), the item type/format (Bridgeman and Cline, 2000), participant motivational and emotional attributes (Sideridis et al., 2014), or even characteristics present in special populations (Sideridis, 2016) may be responsible, for the differential effects of response time on person abilities. Further evidence points to the contribution of and existence of temperamental differences between fast and slow responders (Mayerl, 2013). According to Kennedy (1930): “The slow type is supposed to plod along persistently with great care for details and accuracy. The quick type, …, works in a more slap-dash fashion, has little regard for details, and is inclined to be inaccurate” (p. 286). The results from this study, however, showed the opposite. Fast respondents on difficult items appear to be individuals of high ability, who do not invest additional time, simply because they are confident about their ability to provide the correct answer and provide it quickly. The probability of success for this group is higher than that of slow respondents, who tend to invest additional time but with no apparent success. The quality of time is likely an important factor that should be investigated in observational studies. For example, do low achievers utilize time in an effortful and adaptive manner by being concentrated and focused on the task at hand? Furthermore, whether speededness is an idiosyncratic characteristic, trait or learned behavior (i.e., spontaneous and fast vs. thoughtful and slow respondents) or is simply a matter of ability (i.e., how confident a person is about an answer) is an issue that needs further investigation.

This study has some limitations that need to be pointed out. First, the statistical method used to examine the effect of item response latency on item difficulty, although is a robust method in examining the effect of covariates on both latent factors and factor indicators, it provides estimates only for the thresholds (item difficulties) but not for the slopes (item discriminations). If we are interested in examining the effect of predictors on both, item difficulty and item discrimination parameters, other statistical techniques should be applied, mainly within the IRT framework (e.g., Wang and Hanson, 2005; van der Linden, 2010). A second limitation is that our conclusions are based on a limited array of cognitive ability (i.e., sentence completion, analysis, and critical thinking). Further research is needed to examine the generalizability of current findings with a wider set of cognitive abilities.

Research on item response latency has received great attention lately, especially after the rapid developments on computer-based testing. Previous findings suggest that information regarding item response latency could be used in several different ways, from selecting effective items for CAT, to determine the optimum time limit on tests. However, more studies are needed to examine the roles of both person and item characteristics, and their interaction, toward answering correctly an item. For example, it is of great interest how item response latency is related to different respondent groups (e.g., individuals who utilize a skip pattern, dual responders, etc.), since previous findings have shown that there is a difference in time response between slow and fast respondents (Yang et al., 2002). Thus, an idea would be to examine whether this difference in response latency across different respondent groups is related to the item and person parameterization.

Another idea for future research could be the examination for possible non-linear relationships between item parameters (e.g., item difficulty) and test performance. Recent advances in the investigation of non-linear relationships via cusp catastrophe models, for example, could provide further insights in the interpretation of complicated sets of behavior such as unexpected sudden jumps, where the performance changes unexpectedly at different levels of the ability spectrum. Previous applications of cusp catastrophe model in education and cognitive science have shown the effectiveness of this approach in explaining complex and unexpected patterns of behavior when non-linear relationships exist (e.g., Guastello et al., 2012; Stamovlasis and Tsaparlis, 2012). Finally, by accommodating item response latency in a computerized assessment model, we can elicit valuable information about both, test quality and test takers' behavior, and with that way, enhancing and improving measurement quality and the means to obtain more accurate estimates of person abilities.

## Author contributions

IT and GS contributed to the study conception and design. IT conducted the data analysis and drafted the first version of the manuscript. GS and AA-S edited and provided critical revisions to the manuscript. AA-S provided acquisition to the data, and contributed to the Methods section.

### Conflict of interest statement

The authors declare that the research was conducted in the absence of any commercial or financial relationships that could be construed as a potential conflict of interest.

## Footnotes

^1^The present model deviates markedly from the classical DIF analysis in that items scores are regressed on a single grouping variable (e.g., gender). In the present study, each item was associated with its unique response time pattern so that classifications as slow/fast responders would be item specific.
