# Supplementary material for: An IRT–Multiple Indicators Multiple Causes (MIMIC) Approach as a Method of Examining Item Response Latency
Source: Front Psychol. 2018 Nov 13;9:2177. doi: 10.3389/fpsyg.2018.02177 (PMC6277868; doi:10.3389/fpsyg.2018.02177)
Supplement: Supplementary file 1 [file Data_Sheet_1.PDF]

Supplementary Material

An IRT - Multiple Indicators Multiple Causes (MIMIC) Approach as a Method of Examining Item Response Latency

Ioannis, Tsaousis[1]*, Georgios, D. Sideridis[2,3], Abdullah Al-Sadaawi[4,5]

[1]University of Crete, Department of Psychology, Rethymno, Greece
[2]Boston Children's Hospital, Harvard Medical School, Boston, Massachusetts, USA
[3]National and Kapodistrian University of Athens, Navarinou 13A, Athens, Greece
[4] King Saud University, Riyadh, Saudi Arabia
[5] National Center for Assessment, Riyadh, Saudi Arabia

*** Correspondence:** Ioannis Tsaousis, University of Crete, Department of Psychology, Gallos Campus, Rethymnon, 74100, Greece: tsaousis@uoc.gr

1.1 Supplementary Tables

Table 1

Probability of success and person's estimated ability (in logits) for each item of the Analogy (AN) scale across the two groups of respondents

	Fast Respondents		*Slow Respondents*	
Item	Probability of Success (%)	Person's Ability (in logits)	Probability of Success (%)	Person's Ability (in logits)
1	99	6.13	98	2.76
2	99	2.74	96	1.39
3	99	2.63	90	0.97
4	97	1.46	69	0.35
5	98	1.67	88	0.86
6	89	0.90	61	0.20
7	85	0.74	48	-0.04
8	76	0.49	52	0.04
9	75	0.47	50	0.00
10	74	0.46	74	0.46
11	75	0.47	72	0.41
12	67	0.30	45	-0.09
13	54	0.07	29	-0.39
14	54	0.07	49	-0.02
15	43	-0.13	34	-0.29

Table 2

Probability of success and person's estimated ability (in logits) for each item of the Sentence Completion (SC) scale across the two groups of respondents

	Fast Respondents		*Slow Respondents*	
Item	Probability of Success (%)	Person's Ability (in logits)	Probability of Success (%)	Person's Ability (in logits)
1	99	2.80	88	0.86
2	99	2.37	94	1.18
3	94	1.21	71	0.38
4	85	0.75	78	0.55
5	76	0.51	60	0.17
6	69	0.35	50	0.00

Table 3

Probability of success and person's estimated ability (in logits) for each item of Context Analysis (CA) scale across the two groups of respondents

	Fast Respondents		*Slow Respondents*	
Item	Probability of Success (%)	Person's Ability (in logits)	Probability of Success (%)	Person's Ability (in logits)
1	99	3.78	99	3.47
2	99	2.26	97	1.50
3	96	1.35	86	0.77
4	88	0.87	63	0.23
5	83	0.68	76	0.49
6	79	0.57	36	-0.25
7	79	0.56	63	0.23
8	54	0.06	51	0.02
9	40	-0.18	32	-0.33

Table 4

Probability of success and person's estimated ability (in logits) for each item of Reading Comprehension (RC) scale across the two groups of respondents

	Fast Respondents		*Slow Respondents*	
Item	Probability of Success (%)	Person's Ability (in logits)	Probability of Success (%)	Person's Ability (in logits)
1	99	3.86	99	3.18
2	99	3.17	99	2.76
3	99	3.11	99	2.02
4	95	1.29	97	1.56

5	93	1.09	67	0.32
6	89	0.80	68	0.33
7	76	0.49	58	0.14
8	72	0.42	82	0.65
9	71	0.39	77	0.52
10	65	0.28	50	0.01
11	66	0.28	83	0.68
12	57	0.12	78	0.56
13	50	0.00	40	-0.17
14	39	-0.20	46	-0.06
15	38	-0.21	37	-0.23
16	25	-0.48	61	0.20
17	21	-0.57	54	0.07
18	20	-0.61	33	-0.31

Table 5

Probability of success and person's estimated ability (in logits) for each item of the Arithmetic (AR) scale across the two groups of respondents

	Fast Respondents		*Slow Respondents*	
Item	Probability of Success (%)	Person's Ability (in logits)	Probability of Success (%)	Person's Ability (in logits)
1	92	1.04	85	0.76
2	59	0.16	58	0.14
3	39	-0.19	36	-0.25
4	28	-0.40	22	-0.56
5	27	-0.43	34	0.29
6	25	-0.48	47	-0.05
7	24	-0.51	23	-0.52
8	18	-0.66	17	-0.68
9	17	-0.70	44	-0.11
10	09	-0.98	12	-0.86
11	06	-1.22	11	-0.90
12	01	-2.26	01	-2.28

Table 6

Probability of success and person's estimated ability (in logits) for each item of the Arithmetic Comparisons (CO) scale across the two groups of respondents

	Fast Respondents		*Slow Respondents*	
Item	Probability of Success (%)	Person's Ability (in logits)	Probability of Success (%)	Person's Ability (in logits)
1	99	4.79	99	2.50
2	98	1.61	89	0.93
3	85	0.76	91	1.00
4	75	0.49	74	0.45
5	39	-0.19	52	0.03
6	05	-1.27	13	-0.82

Table 7

Probability of success and person's estimated ability (in logits) for each item of the Critical Thinking (CT) scale across the two groups of respondents

	Fast Respondents		*Slow Respondents*	
Item	Probability of Success (%)	Person's Ability (in logits)	Probability of Success (%)	Person's Ability (in logits)
1	99	3.78	99	2.79
2	99	3.29	99	2.46
3	99	3.01	97	1.47
4	92	1.07	80	0.61
5	94	1.18	93	1.15
6	92	1.04	82	0.65
7	71	0.38	70	0.37
8	65	0.27	75	0.47
9	61	0.19	71	0.39
10	44	-0.11	20	-0.60
11	42	-0.13	49	-0.02
12	40	-0.17	20	-0.60

Table 8

Probability of success and person's estimated ability (in logits) for each item of the Spatial (SP) scale across the two groups of respondents

	Fast Respondents		*Slow Respondents*	
Item	Probability of Success (%)	Person's Ability (in logits)	Probability of Success (%)	Person's Ability (in logits)
1	99	12.40	99	12.39
2	99	4.68	99	4.68
3	99	2.98	99	2.15
4	99	3.50	99	3.50
5	97	1.51	98	1.72
6	83	0.70	67	0.31
7	62	0.21	68	0.33
8	34	-0.28	15	-0.74
9	19	-0.63	40	-0.17
10	08	-1.06	22	-0.55

Table 9

Probability of success and person's estimated ability (in logits) for each item of the Logic (LG) scale across the two groups of respondents

	Fast Respondents		*Slow Respondents*	
Item	Probability of Success (%)	Person's Ability (in logits)	Probability of Success (%)	Person's Ability (in logits)
1	67	0.31	82	0.65
2	67	0.31	55	0.09
3	66	0.29	64	0.25
4	48	-0.03	51	0.01
5	35	-0.28	71	0.39
6	24	-0.50	11	-0.91
7	22	-0.54	49	-0.02
8	14	-0.80	33	-0.30
9	12	-0.85	88	0.85
10	08	-1.08	09	-1.00

1.2 Figures

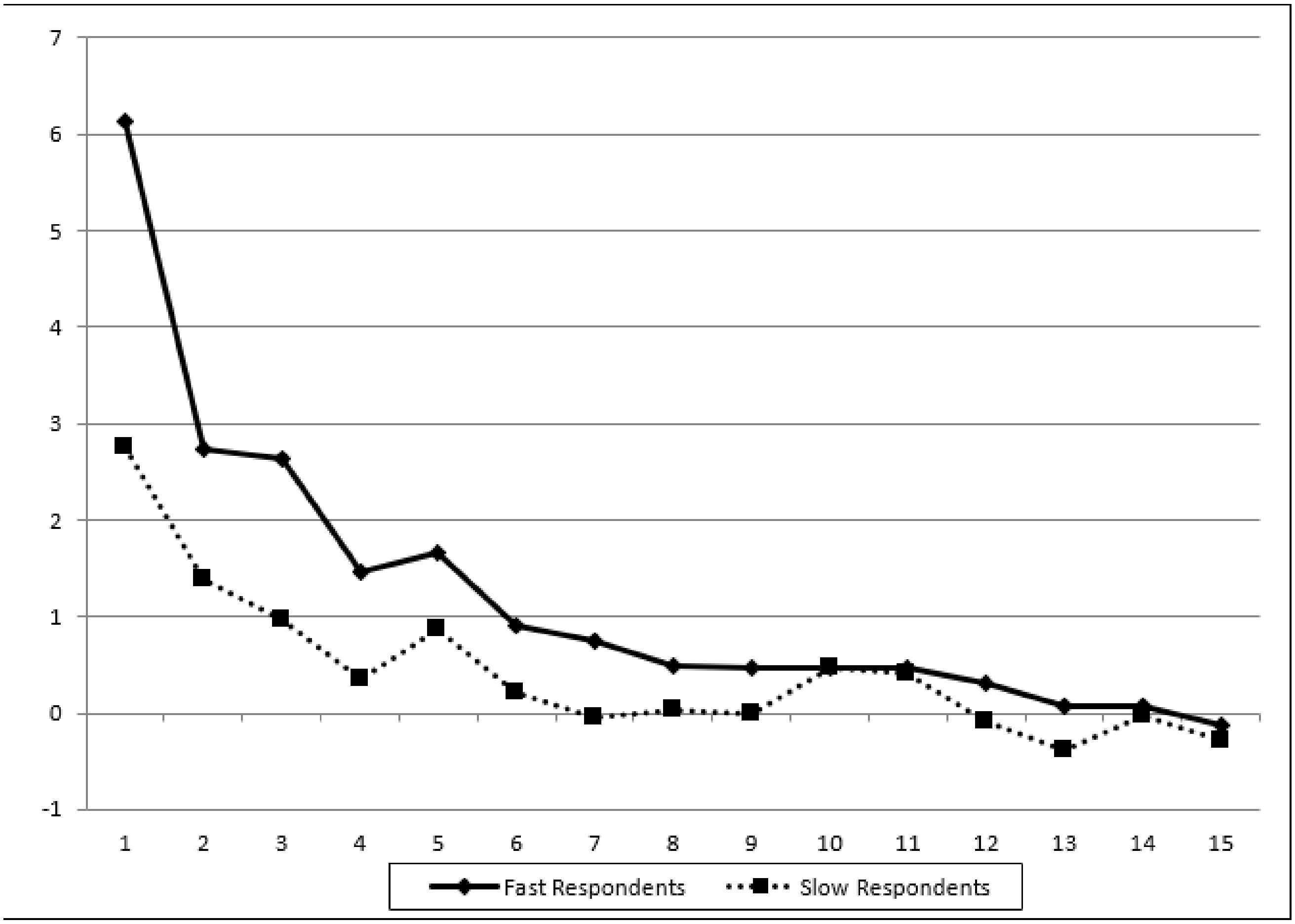

Supplementary Figure 1. Person's estimated ability (in logits) across different groups of respondents (fast vs. slow) for the Analogy (AN) scale

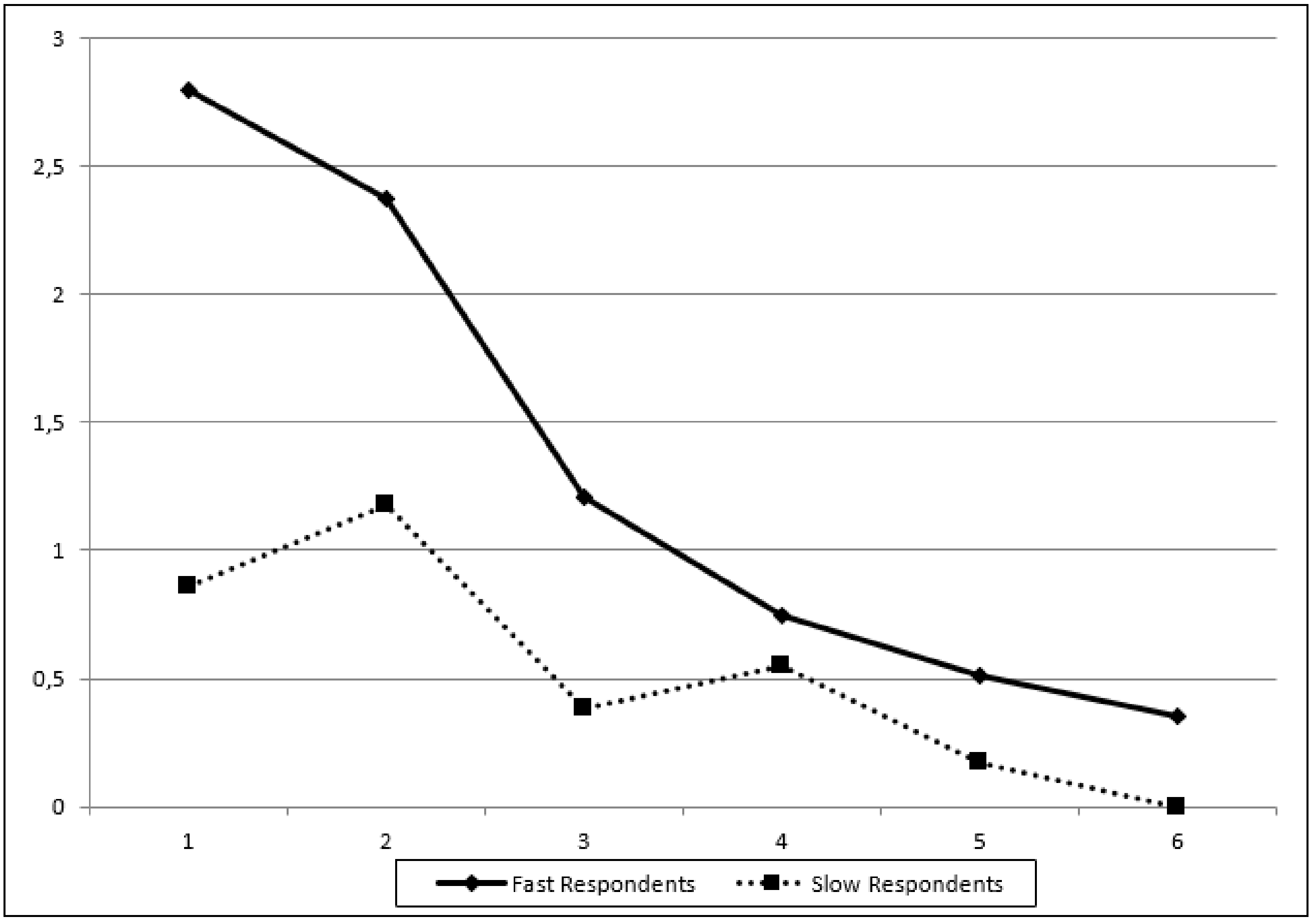

Supplementary Figure 2. Person's estimated ability (in logits) across different groups of respondents (fast vs. slow) for the Sentence Completion (SC) scale

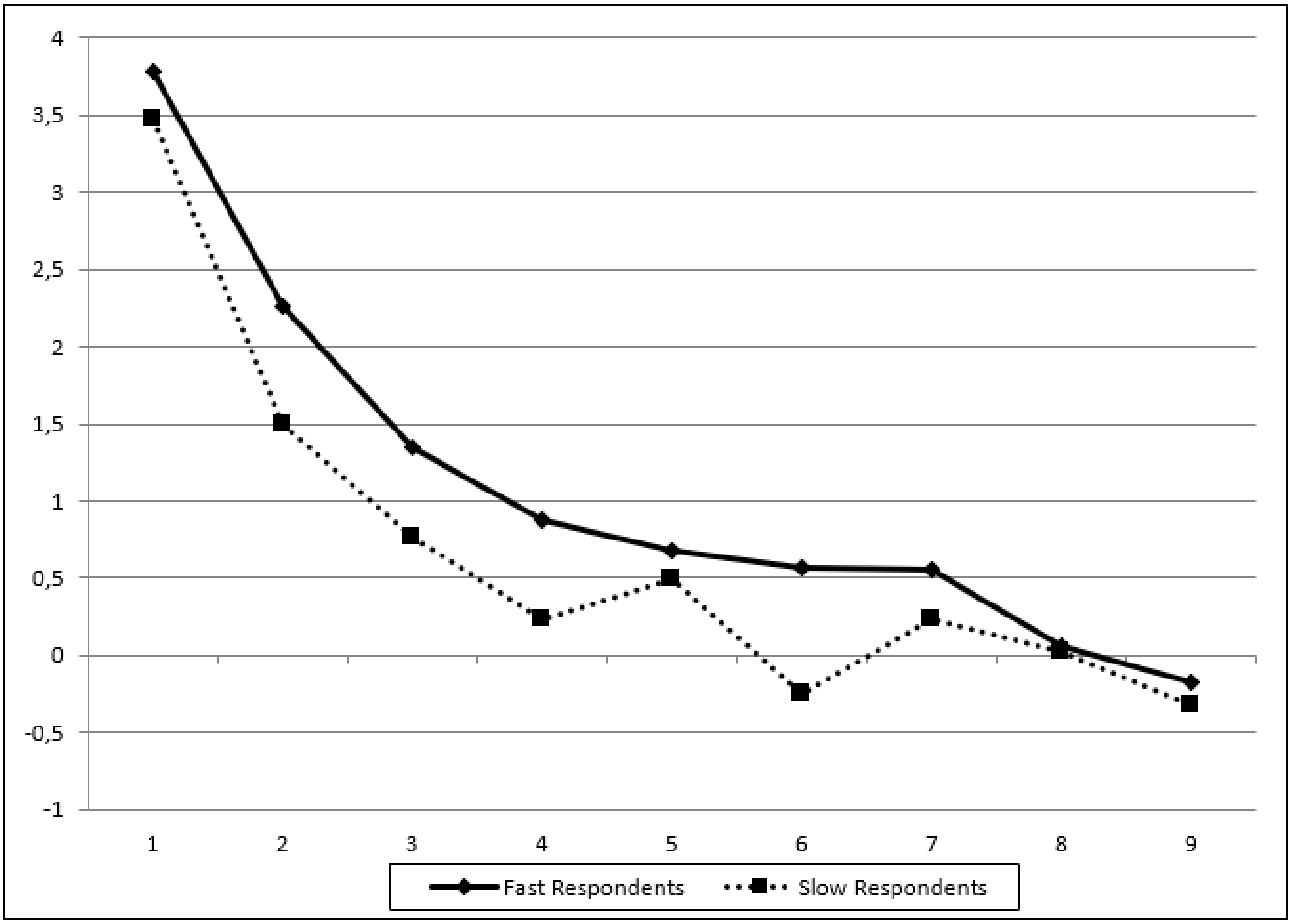

Supplementary Figure 3. Person's estimated ability (in logits) across different groups of respondents (fast vs. slow) for the Sentence Context Analysis (CA) scale

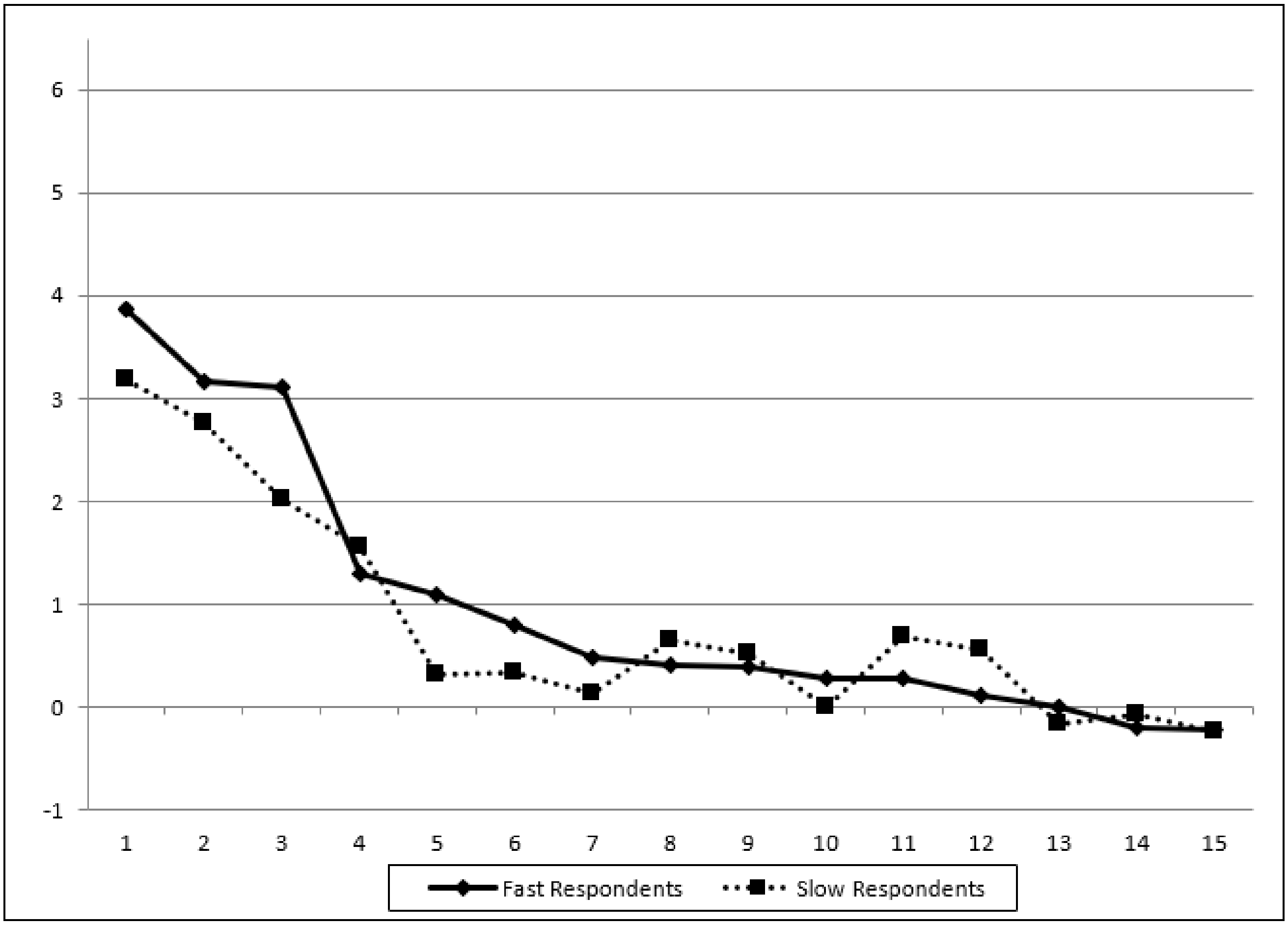

Supplementary Figure 4. Person's estimated ability (in logits) across different groups of respondents (fast vs. slow) for the Reading Comprehension (RC) scale

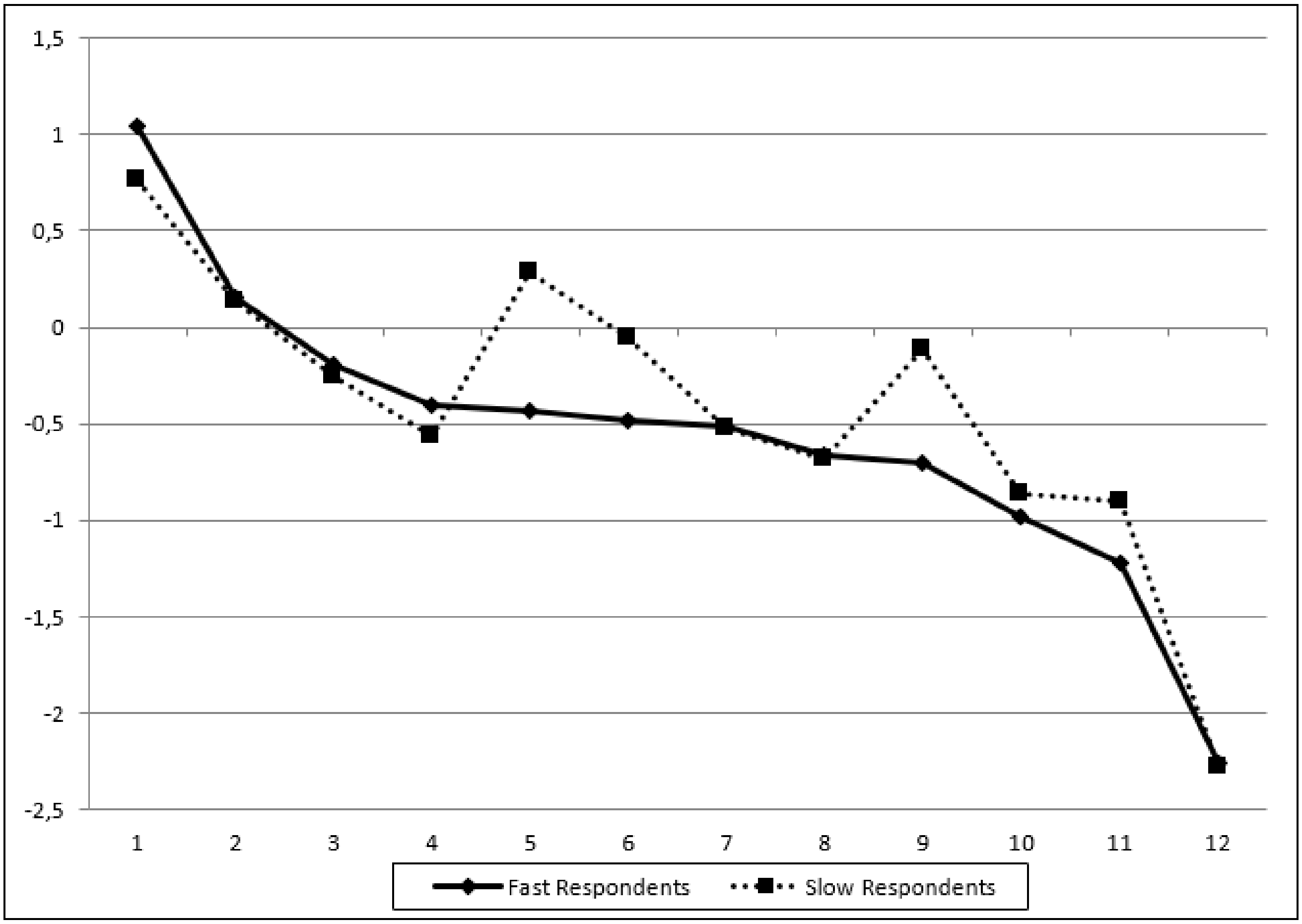

Supplementary Figure 5. Person's estimated ability (in logits) across different groups of respondents (fast vs. slow) for the Arithmetic (AR) scale

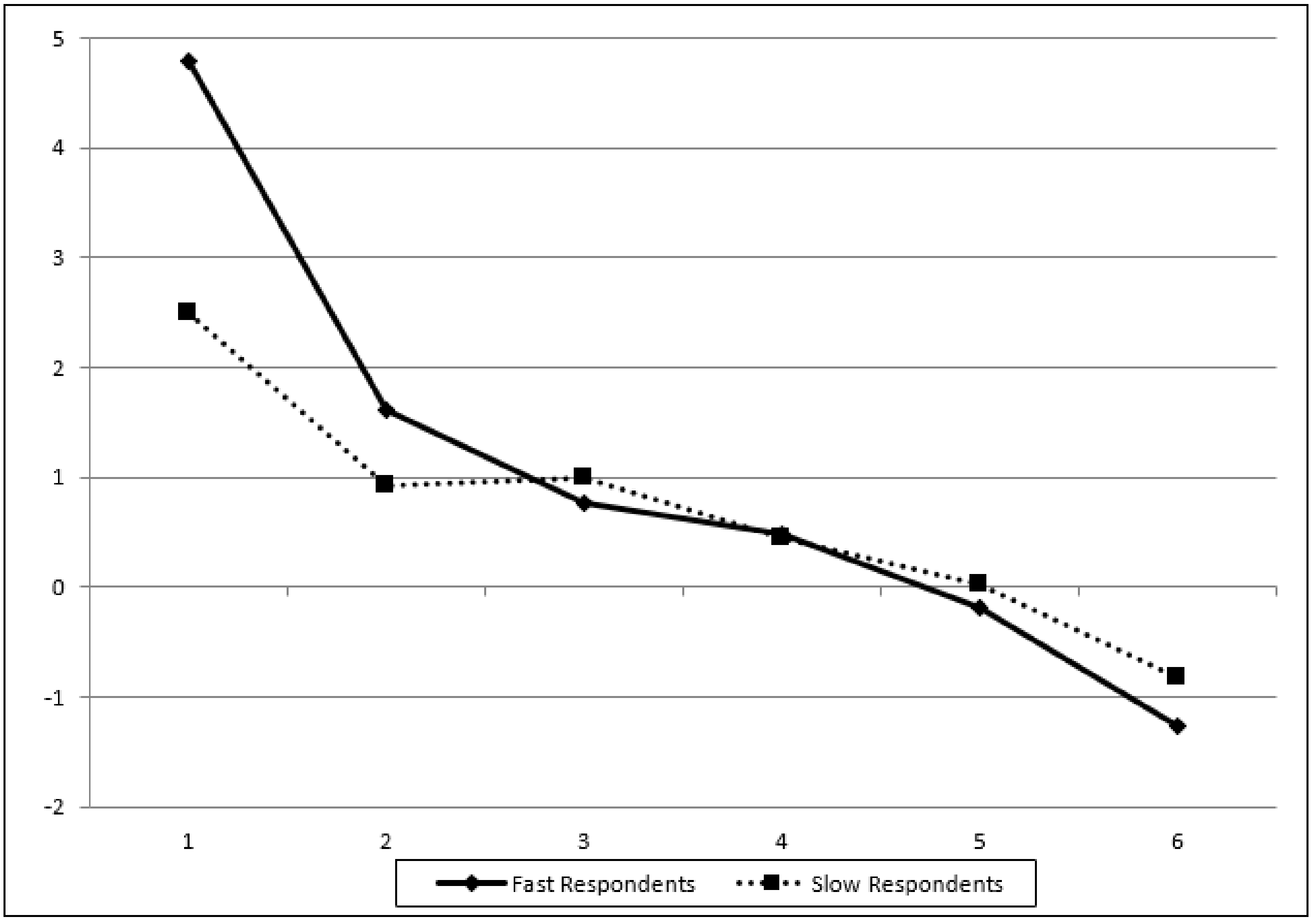

Supplementary Figure 6. Person's estimated ability (in logits) across different groups of respondents (fast vs. slow) for the Arithmetic Comparisons (CO) scale

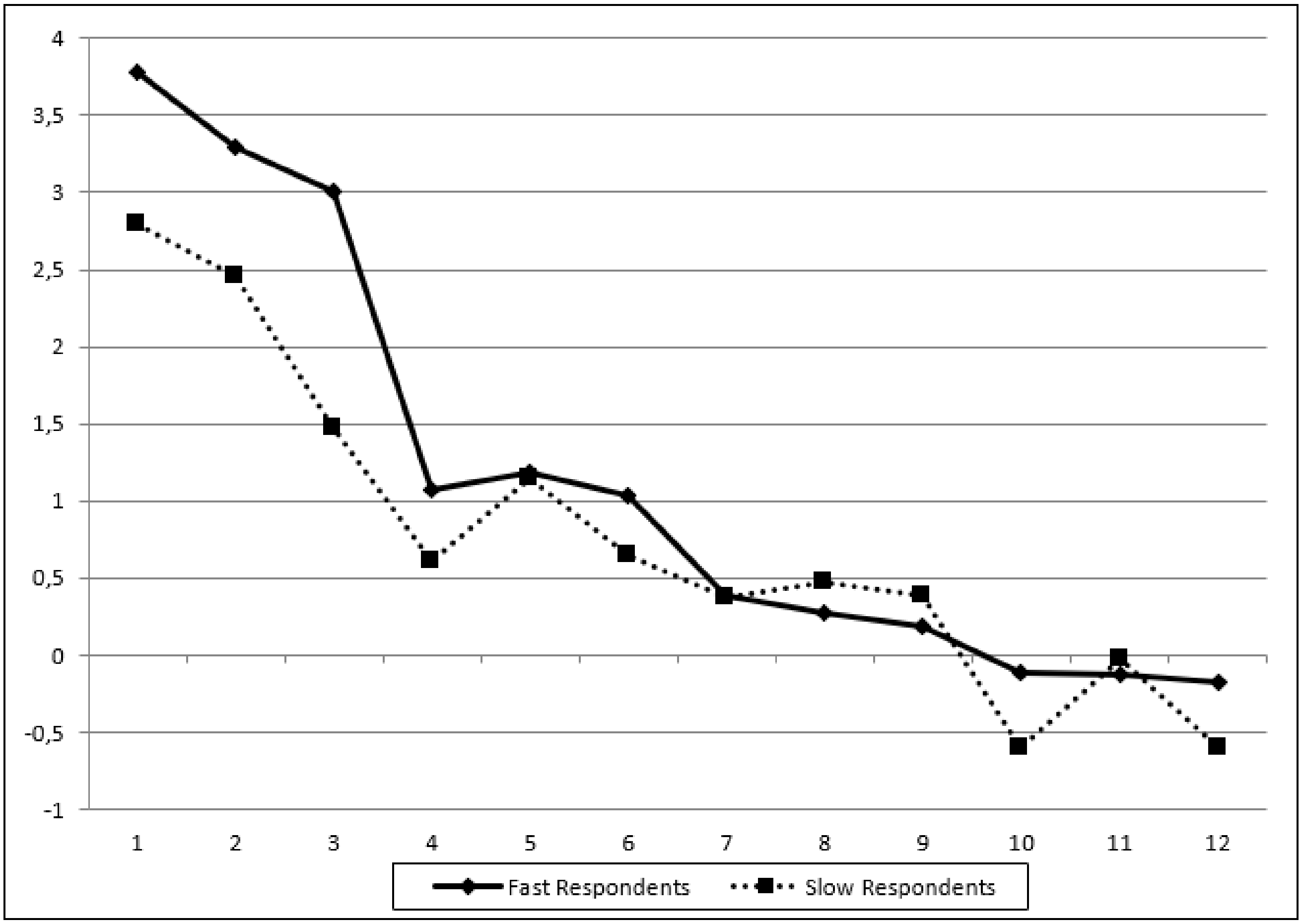

Supplementary Figure 7. Person's estimated ability (in logits) across different groups of respondents (fast vs. slow) for the Critical Thinking (CT) scale

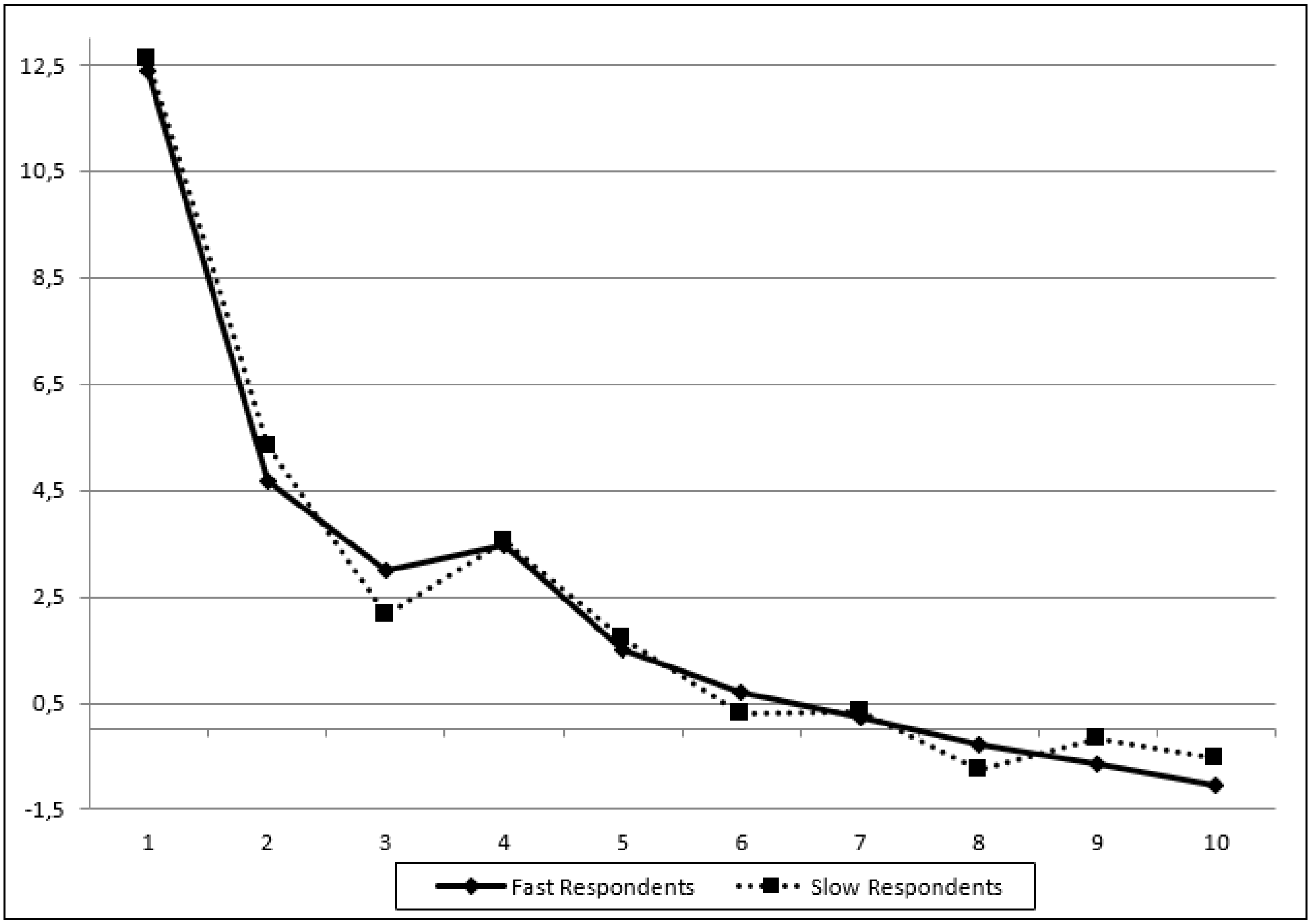

Supplementary Figure 8. Person's estimated ability (in logits) across different groups of respondents (fast vs. slow) for the Spatial (SP) scale

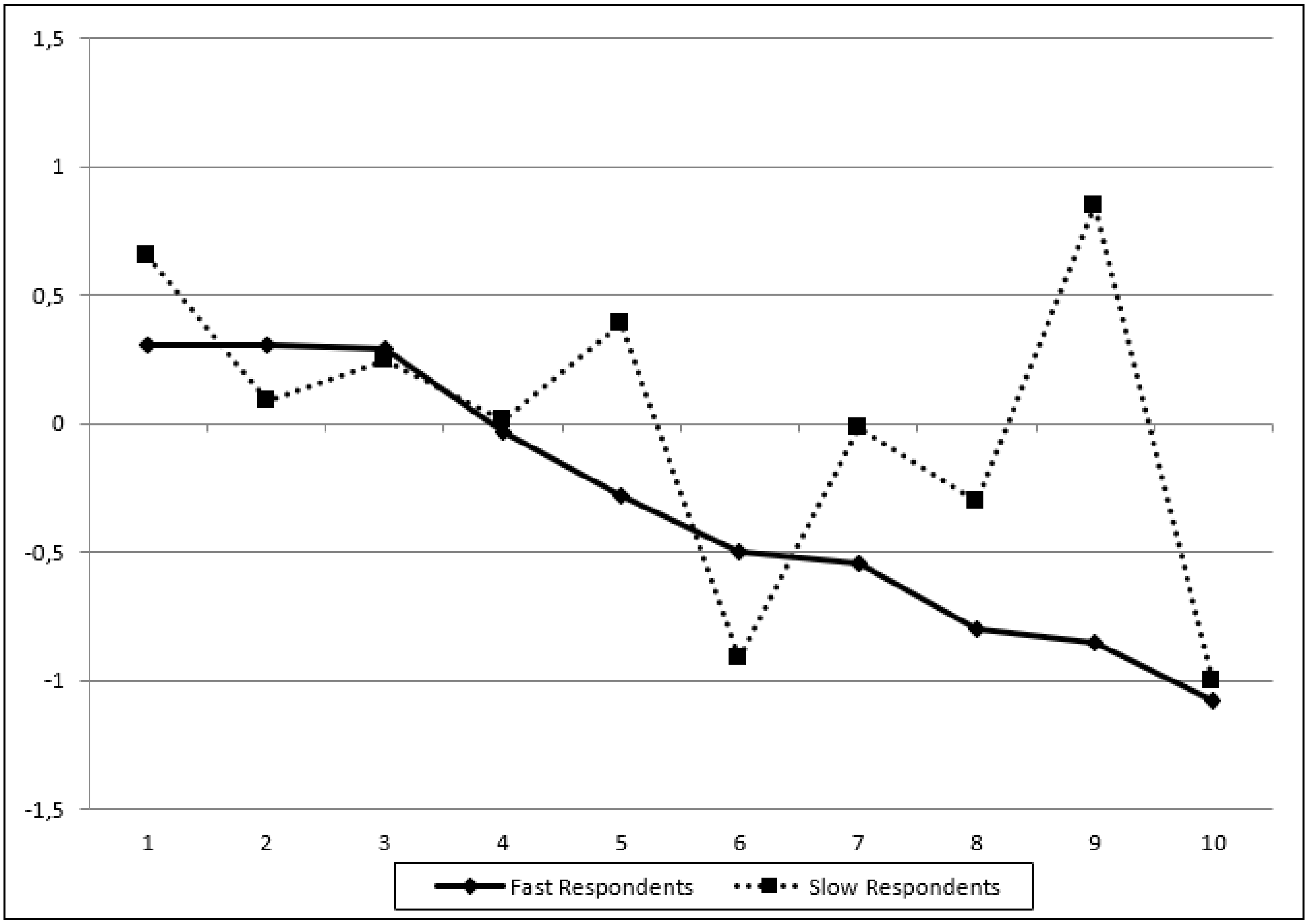

Supplementary Figure 9. Person's estimated ability (in logits) across different groups of respondents (fast vs. slow) for the Logic (LG) scale
